# Factor VIII inhibitor bypass activity (FEIBA) for the reduction of transfusion in cardiac surgery: a randomized, double-blind, placebo-controlled, pilot trial

**DOI:** 10.1186/s40814-021-00873-5

**Published:** 2021-07-02

**Authors:** Valerie A. Sera, Ann E. Stevens, Howard K. Song, Victor M. Rodriguez, Frederick A. Tibayan, Miriam M. Treggiari

**Affiliations:** 1 Department of Anesthesiology and Perioperative Medicine, Oregon Health & Science University, Portland, OR USA; 2 Department of Anesthesiology, Kaiser Permanente, Portland, OR USA; 3 Department of Cardiothoracic Surgery, Oregon Health & Science University, Portland, OR USA; 4 Department of Surgery, Division of Cardiothoracic Surgery, UC Davis Vascular Center, Davis, CA USA; 5 Department of Anesthesiology, Yale University, 333 Cedar Street, TMP-3, New Haven, CT USA

**Keywords:** Adult anesthesia, Anesthetics, Cardiac surgery, Bleeding disorders, Coagulopathies, Hematology

## Abstract

**Background:**

Uncontrolled bleeding after cardiac surgery can be life-threatening. Factor eight inhibitor bypassing activity (FEIBA) is a prothrombin complex concentrate empirically used as rescue therapy for correction of refractory bleeding diathesis post-cardiopulmonary bypass (CPB). FEIBA used as rescue therapy for bleeding diathesis after CPB has been associated with a low incidence of complications and a reduction in transfusion requirement and re-exploration. The feasibility and efficacy of early administration of FEIBA after the termination of CPB have not been studied in a prospective randomized trial.

**Methods:**

We designed a small randomized, double-blinded, placebo-controlled pilot trial to determine the feasibility of a larger trial testing the hypothesis that FEIBA decreases transfusion requirements after CPB. The study was designed to evaluate the feasibility of a larger pivotal trial to determine the effectiveness of FEIBA in reducing the total volume of blood products transfused perioperatively, and its safety profile. Study participants were adult patients undergoing elective major aortic cardiovascular surgery at a tertiary referral hospital, who were equally randomized to receive a single dose of either FEIBA or matched placebo intraoperatively at the end of CPB.

**Results:**

Twenty patients were screened and 12 were randomized and included in the analysis. Protocol adherence was high, and all patients received the study drug per intention-to-treat except one patient. There were no protocol deviations or events of unblinding, and adverse events were not different between groups. Patients in the FEIBA group were older and more likely to be female and had higher BMI, lower hematocrit, and longer hypothermic circulatory arrest. There were no differences in post-randomization blood product transfusions (difference FEIBA vs. placebo −899 mL; 95% CI −5206 to 3409) or in the administration of open-label FEIBA.

**Conclusions:**

This pilot trial confirmed the adequacy of the trial design that involved the early, blinded administration of FEIBA, by demonstrating excellent protocol adherence. We conclude that a larger trial establishing the effectiveness of early prothrombin complex concentrate administration to reduce the use of blood products in the setting of high-risk cardiac surgery is feasible.

**Trial registration:**

ClinicalTrials.gov, NCT02577614. Registered 16 October 2015

## Key messages

- What uncertainties existed regarding the feasibility?

FEIBA used as rescue therapy for bleeding diathesis after CPB has been associated with a low incidence of complications and a reduction in transfusion requirement and re-exploration. However, FEIBA has not been prospectively investigated as an early treatment for the reduction of transfusion requirements.

- What are the key feasibility findings?

There were no protocol deviations or events of unblinding, and adverse events were not different between groups. There were no differences in post-randomization blood product transfusions or in the administration of open-label FEIBA.

- What are the implications of the feasibility findings for the design of the main study?

A larger trial establishing the effectiveness of early prothrombin complex concentrate administration to reduce the use of blood products in the setting of a high-risk cardiac surgery is feasible.

## Background

It is recognized that blood product transfusion has been associated with adverse outcomes in cardiac surgery, including increased risk of infection, hospital length of stay, and mortality. While cardiac surgeons and anesthesiologists appreciate the risk of morbidity and mortality with blood product transfusion [1–7], it is challenging to find alternative strategies to correct bleeding diathesis after cardiopulmonary bypass (CPB). Current prophylactic management of CPB-associated bleeding diathesis is by infusion of antifibrinolytic medications, such as tranexamic acid and ε-aminocaproic acid. However, refractory bleeding still occurs despite the use of antifibrinolytics.

In cardiac surgical procedures at high risk for bleeding after separation from CPB such as those involving the aorta with long CPB and aortic cross-clamp times, refractory bleeding diathesis is commonly managed with the initiation of rescue therapy with factor concentrates. The evidence for prothrombin complex concentrate products, albeit limited, suggests decreased intraoperative transfusion requirements [8, 9]. However, there are also substantial risks which include stroke or other thrombotic events which may be life-threatening [10].

Factor eight inhibitor bypassing activity (FEIBA) is principally composed of the clotting factors of the prothrombin complex, chiefly factors II, VII, IX, and X. Whereas in licensed prothrombin complex concentrates (PCCs), the coagulation factors are present as zymogens, FEIBA does contain small amounts of activated coagulation factors, in addition to the zymogens [11, 12]. Furthermore, PCCs contain proteins C and S as a safeguard against undesired coagulation activation, plus added heparin/antithrombin complex. PCCs are essentially used to replace a deficit in clotting factors, whereas FEIBA is designed to directly trigger the clotting process. The mechanism of action is linked to the prothrombinase complex and to the interaction between prothrombin (F II) and activated factor X (F Xa). FEIBA is currently approved for use in the USA for the treatment of patients with hemophilia and inhibitors at a dose of 50–100 IU/kg. There is some evidence demonstrating the safety and efficacy of FEIBA for hemophilia patients with inhibitors [11, 13], as well as for the reversal of warfarin, dabigatran, rivaroxaban, and other anticoagulation products [12, 14, 15]. FEIBA has a theoretical advantage compared to activated factor VII in that it replenishes multiple depleted factors that are lost with CPB. This factor replenishment with FEIBA may lead to improved hemostasis, possibly with lower thrombotic risk compared to activated factor VII [1–4, 10, 13–20].

Our institution has experience with the use of FEIBA for the rescue treatment of CPB-associated coagulopathy. In a retrospective study of 25 high-risk patients that received FEIBA as rescue therapy for post-CPB bleeding diathesis using a conservative dose of 10–25 IU/kg (average of 2100 IU total dose per patient), we found that the use of fresh-frozen plasma and platelet transfusion after FEIBA administration was significantly lower compared to the amount of blood products transfused prior to FEIBA. No patients were returned to the operating room for re-exploration for bleeding. One patient developed an upper extremity deep vein thrombosis in the setting of central venous catheterization [18].

We conducted a pilot study to evaluate the feasibility of the prophylactic administration of FEIBA after the termination of CPB and a reversal dose of IV protamine sulfate. The study was designed to demonstrate the feasibility of a trial investigating the potential role of FEIBA administration in reducing the need for allogeneic transfusion to treat refractory bleeding diathesis in patients undergoing a high-risk cardiovascular surgery.

## Methods

### Study design

We conducted a single-center, double-blind, placebo-controlled, randomized pilot trial to assess the feasibility and safety of prophylactic FEIBA administration in patients undergoing elective major aortic cardiovascular surgery requiring CPB. The study was conducted at the Oregon Health and Science University adult cardiac operating rooms between August 1, 2016, and August 31, 2017. The study protocol was approved by the Institutional Review Board. Patients were enrolled in the trial after providing written informed consent preoperatively. The study adheres to CONSORT guidelines.

### Study population

Patients were eligible if they were adults 18 years or older, scheduled for elective aortic procedures, including ascending, arch, and descending repair or reconstruction, with cardiopulmonary bypass, aortic valve repair or replacement, coronary re-implantation (Bentall), and/or deep hypothermic circulatory arrest. Patients were excluded if they were unable to receive the study drug based on contraindications stated by the manufacturer, such as known anaphylactic or severe hypersensitivity reactions to FEIBA or any of its components or received a blood transfusion within 28 days. Patients were also excluded if their scheduled procedure included coronary artery bypass grafting; had a history of myocardial infarction, thrombosis, or embolism; disseminated intravascular coagulation; or were pregnant women, decisionally impaired, prisoners, or unwilling to provide informed consent.

### Randomization and blinding

A computer-generated random list using a uniform distribution to equally (fair-coin randomization) assign patients to either FEIBA or placebo was created and maintained by the investigational pharmacy. The investigational pharmacy prepared a study drug that was delivered to the providers in the operating room by an anesthesia technician. Patients, surgeons, anesthesiologists, and nurses were blinded to treatment assignment. The research pharmacy prepared the FEIBA (at the concentration of 40 IU/mL) or placebo (normal saline) in an opaque syringe. To maintain blinding, the volume of FEIBA and the matched placebo were prepared based on a milliliter per kilogram of actual body weight. Blinded assessors collected data on primary and secondary endpoints, including transfusion requirements, chest tube drainage, safety endpoints, and protocol adherence auditing.

### Study groups

After anesthesia induction, patients were equally randomized by the research coordinator to receive either a single dose of FEIBA or a matched volume of saline administered after separation from CPB. The active study drug was prepared in the dose of 20 IU per kilogram in a concentration of 40 IU/mL, at a rate of 0.5 mL/kg via infusion pump over 10 min. The placebo consisted of a matched volume of 0.9% sodium chloride supplied in an identical syringe and tubing at a rate of 0.5 mL/kg via infusion pump over 10 min. Patients were otherwise managed per usual care according to a standardized protocol.

### Study procedures

**Fig. 1:**
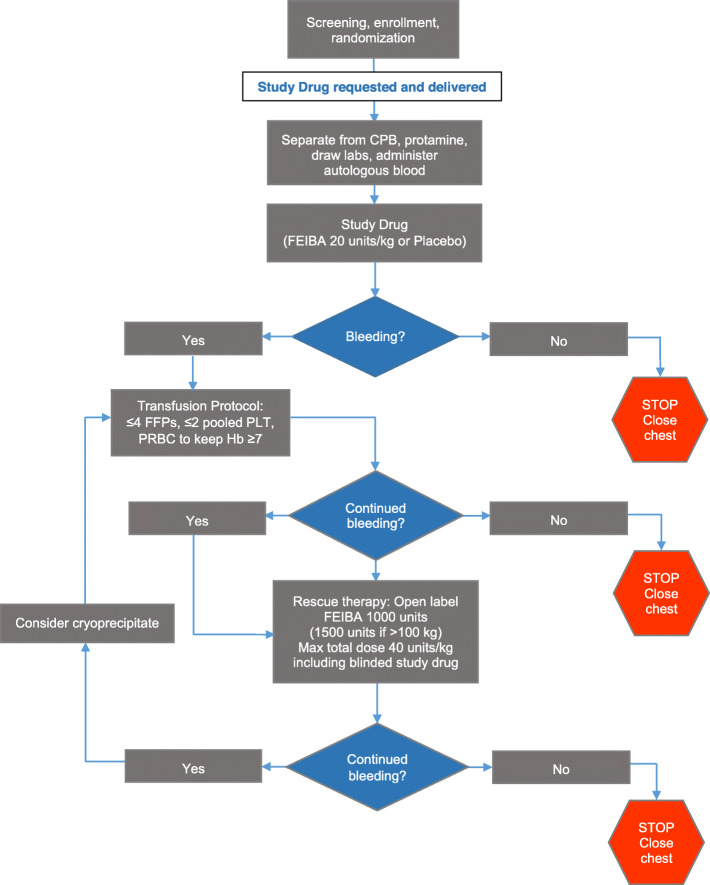
Study design and decision algorithm to guide the management of bleeding diathesis after separation from cardiopulmonary bypass (CPB). FEIBA, factor VIII inhibitor bypass activity; FFPs, fresh-frozen plasma; PLT, platelet; PRBCs, packed red blood cells

After separation from CPB, a reversal dose of intravenous protamine sulfate, calculated according to the heparin dose-response curve, was given with a target goal of a heparin concentration of zero or a return to baseline activated clotting time (ACT). As part of our standard care, routine post-CPB labs, a complete blood count, and coagulopathy panel including the hemoglobin, hematocrit, platelet count, INR, aPTT, and fibrinogen were sent after the administration of IV protamine immediately following separation from CPB, with a post-protamine ACT and arterial blood gas. In addition, samples were collected for thromboelastogram (TEG) by the research lab. After ACT normalized and labs were drawn, the study drug was administered, and the field was subsequently inspected for ongoing microvascular bleeding. In the presence of refractory bleeding diathesis, the anesthesiologist was permitted to administer a dose of FEIBA in an open-label manner as a rescue measure based on the algorithm described below and illustrated in Fig. 1. This was a shared decision by the cardiac surgeon and anesthesiologist attending. However, the treating team remained blinded to the earlier, prophylactic administration of the study drug. Patients who experienced hemorrhage received, as the first line, standard therapy with blood products including the fresh-frozen plasma (FFP), platelets, and packed red blood cells (PRBCs). Up to 1 apheresis platelets and 2 FFPs could be administered empirically, then additional products were administered based on the coagulation panel (target INR ≤1.7 and aPTT ≤50 s). PRBCs were administered with the goal of maintaining a hemoglobin level ≥7 mg/dL. If refractory bleeding persisted after the empiric or laboratory-based administration of at least 4 units of FFPs and 2 apheresis platelets, then rescue therapy of 10–20 IU/kg (or 1500 units for patients >150 kg) of open-label FEIBA was given to patients who displayed refractory bleeding diathesis. Subsequently, after the administration of FEIBA, cryoprecipitate and lab-guided administration were continued. The study was designed such that the total dose of FEIBA would not exceed 40 IU/kg (including the presumed 20 IU/kg given as a study drug). The supply of FEIBA for open-label use was prepared by the anesthesiologist according to manufacturer recommendations.

### Sample size

We planned to enroll 12 patients for the initial feasibility and safety study. As this study was designed to evaluate the feasibility and safety of FEIBA administration in the setting of high-risk cardiovascular surgery with CPB, it did not have adequate power for efficacy endpoints.

### Study endpoints

The primary goal of this pilot trial was to document the feasibility of the prophylactic blinded administration of FEIBA in the context of cardiovascular procedures at high risk of coagulopathy and requirement for blood product transfusion. Indicators of feasibility were protocol violations, maintenance of blinding, use of open-label FEIBA, and occurrence of serious adverse events. The primary endpoints for a larger pivotal trial would be a cumulative volume of blood products transfused including the packed red blood cells (PRBCs), fresh-frozen plasma (FFP), platelets, and use of cryoprecipitate, after the administration of the study drug until the end of anesthesia, and the evaluation of the safety profile.

### Statistical analysis

The study was analyzed using a modified intention-to-treat approach. Descriptive summaries are presented using means and standard deviations (SD) for quantitative characteristics and frequencies (%) for categorical characteristics. Data were tested for normality and summaries reported accordingly. Since the study design was randomized, we tested for treatment differences using Welch’s t-tests for mean comparisons of quantitative characteristics and chi-square tests of associations for binary or categorical characteristics. Welch’s t-test was used to test for a treatment effect for the primary endpoint, volume in mL/kg of actual body weight of any blood products transfused after randomization, and for the secondary endpoints. There were no plans for interim analysis; however, safety data was monitored on an ongoing basis during the study. All hypothesis tests evaluated were two-sided, and all analyses were conducted using the Stata (version 15.1) statistical package. The same statistical package was used to create the random sequence for the fair-coin randomization assignment. A two-sided alpha value of 0.05 was required for statistical significance.

## Results

### Demographic and baseline characteristics

**Fig. 2:**
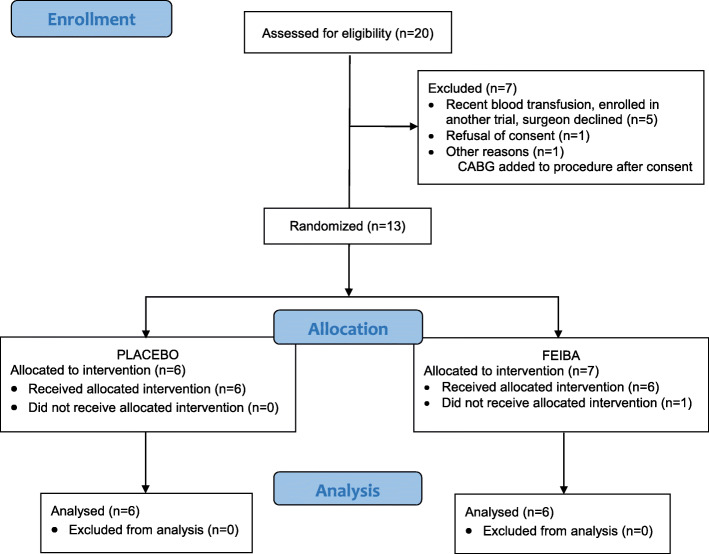
Study flowchart

Twenty patients were screened for eligibility and 13 patients were randomized. Six were allocated to the placebo group and 7 were allocated to the FEIBA group (Fig. 2). One subject in the FEIBA group was randomized in error, did not receive a study drug, and was excluded. Subject baseline characteristics are shown in Table 1, demonstrating unbalances between the FEIBA and the placebo groups in some patients’ demographics, including mean age (63 years versus 49 years) and mean BMI (33 versus 26). Furthermore, the FEIBA group had more females (67% versus 0%) and more patients with hypertension (100% versus 50%) compared to the placebo group. The hematocrit was lower in the FEIBA group compared to the placebo group (39% vs 43%). All other baseline characteristics were similar between groups.

**Table 1:** Patients’ baseline characteristics stratified by treatment assignment. Data are expressed as mean (SD), unless otherwise specified

	FEIBA n = 6	Placebo n = 6
Age, years	62.5 (4.3)	49.2 (13.1)
Gender, n (% female)	4 (67)	0 (0)
Weight, kg	101 (20.0)	84 (9.5)
Height, cm	175 (7.6)	180 (8.4)
Body mass index, kg/cm^2^	33 (5.0)	26 (4.4)
Non-Hispanic, n (%)	6 (100)	5 (83)
Diabetes, n (%)	0 (0)	1 (17)
Hypertension, n (%)	6 (100)	3 (50)
Smoking status, n (%)	3 (50)	2 (33)
CVA, n (%)	2 (33)	0 (0)
Anti-platelet therapy, n (%)	4 (67)	2 (33)
ASA score ≥ 4, n (%)	6 (100)	4 (67)
Baseline labs		
BUN	20 (9.4)	19 (2.8)
Creatinine (mg/dL)	0.89 (0.3)	1.13 (0.2)
Hematocrit (%)	39 (1.7)	43 (2.5)
Platelet count (K/cu)	203 (62)	194 (16)
aPTT	44 (34)	27 (3)
INR	1.05 (0.1)	1.01 (0)
Fibrinogen (mg/dL)	403 (63)	337 (100)

*FEIBA*, factor eight inhibitor bypass activity; *CVA*, cerebrovascular accident; *ASA*, American Society of Anesthesiology physical status; *BUN*, blood urea nitrogen; *aPTT*, activated partial thromboplastin time; *INR*, international normalized ratio

Intraoperative characteristics are shown in Table 2. The mean duration of deep hypothermic cardiac arrest (93 min versus 24 min) was longer in the FEIBA group compared to the placebo group. All other intraoperative characteristics were similar between groups.

**Table 2:** Intraoperative characteristics stratified by treatment assignment, mean (SD)

	FEIBA n = 6	Placebo n = 6
Cardiopulmonary bypass duration, min	276 (111)	170 (53)
Aortic cross-clamp time, min	159 (75)	123 (51)
Lowest temperature during CPB, °C	25 (4)	25 (4)
Deep hypothermic cardiac arrest, n (%)	5 (83)	3 (50)
Deep hypothermic cardiac arrest duration, min	93 (26)	24 (5)
FEIBA study drug dose, IU, mean (SD)	1902 (499)	0
Open-label FEIBA, n (%)	2 (33)	1 (17)
Dose, IU, mean (SD; n1 = 2; n2 = 1)	3772 (1002)	1840 (--)
Dose, IU, mean (SD; n1 = 6; n2 = 6)	1241 (2024)	307 (751)

*FEIBA*, factor eight inhibitor bypass activity; *CPB*, cardiopulmonary bypass

### Study endpoints

During the study implementation, protocol adherence was high, without protocol violations, with the exception of one patient randomized in error. There were no reports of drug administration events requiring troubleshooting that would have unmasked providers as to which study drug was delivered. Likewise, there were no events requiring unblinding.

The administration of open-label FEIBA was not different between the two groups. Additional study endpoints to estimate variability for the planning of a larger trial are shown in Table 3. There was a substantial variability but no difference in the endpoint of the total volume of blood product transfused post-randomization (difference between FEIBA and placebo group −899 mL (95% CI −5205.7 to 3408.7)). Likewise, there were no differences in the individual blood components or in the amount of post-randomization blood product transfused intraoperatively or in the ICU. There were no differences in TEG values before and after study drug administration and between the two groups (Table 4). The volume of chest tube drainage, duration of intubation, and hospital length of stay were not different between the FEIBA and placebo groups (Table 3).

**Table 3:** Study primary and secondary endpoints. Data are expressed as mean (SD), unless otherwise specified

Variable	FEIBA n = 6	Placebo n = 6	Difference (95% confidence interval)
Volume of all blood products transfused, mL	3126 (3710)	2227 (3710)	−899 (−5205.7 to 3408.7)
Blood product transfused intraoperatively			
Packed red blood cells, n (%)	4 (67)	0 (0)	
Packed red blood cells, mL	572 (552)	0 (0)	−572 (−1073.7 to −69.6)
Fresh-frozen plasma, n (%)	4 (67)	4 (67)	
Fresh-frozen plasma, mL	981 (857)	338 (288)	−643 (−1465.2 to 179.2)
Platelets, n (%)	6 (100)	5 (83)	
Platelets, mL	952 (511)	437 (308)	−515 (−1057.2 to 27.2)
Cryoprecipitate, n (%)	3 (50)	0 (0)	
Cryoprecipitate, mL	171 (210)	0	−171 (−362.2 to 20.2)
Blood product transfused in the ICU			
Packed red blood cells, n (%)	1 (17)	1 (17)	
Packed red blood cells, mL	175 (429)	572 (1400)	397 (−935.4 to 1728.8)
Fresh-frozen plasma, n (%)	1 (17)	1 (17)	
Fresh-frozen plasma, mL	102 (250)	437 (1070)	335 (−664.7 to 1333.7)
Platelets, n (%)	1 (17)	1 (17)	
Platelets, mL	49 (119)	83 (204)	35 (−179.8 to 249.2)
Cryoprecipitate, n (%)	1 (17)	1 (17)	
Cryoprecipitate, mL	21 (51)	79 (194)	58 (−123.9 to 240.2)
Patients requiring re-exploration, n (%)	1 (17)	1 (17)	
Chest tube drainage at 8 h, mL	282 (110)	611 (860)	329 (−459.3 to 1117.3)
Chest tube drainage at 24 h, mL	725 (559)	913 (1028)	188 (−876.1 to 1252.4)
ICU intubation, hours	87.2 (162.6)	8.2 (6.2)	−79 (−249.6 to 91.6)
Length of ICU stay, hours	333.5 (670.7)	74.3 (51.1)	259.2 (−962.7 to 444.4)
Length of hospital stay, days	20.3 (25.7)	5.7 (1.0)	14.7 (−41.6 to 12.3)

*FEIBA*, factor eight inhibitor bypass activity; *ICU*, intensive care unit

**Table 4:** Pre- and post-study drug hematological and coagulation profile. Data are expressed as mean (SD), unless otherwise specified

	Prior to study drug		Post-study drug	
	FEIBA n = 6	Placebo n = 6	FEIBA n = 6	Placebo n = 6
R value, min	7.78 (1.83)	6.63 (1.75)	5.5 (1.29)	5.17 (1.27)
K, min	2.34 (1.99)	2.35 (0.69)	2.58 (1.88)	2.12 (1.21)
*α* angle, degrees	66 (15)	61 (7)	65 (12)	63 (12)
MA, mm	53 (13)	55 (6)	55 (12)	60 (6)
A, mm	53 (12)	55 (5.2)	55 (13)	59 (5)
CI	−2.3 (3.9)	−1.6 (2.3)	0.32 (3.0)	0.17 (2.8)
LY 30	0.2 (0.3)	0.03 (0.1)	0.04 (0.05)	0.13 (0.33)
Hematocrit	27.3 (25.0)	27.7 (24.4)	28.6 (22.8)	29.7 (22.4)
Platelets	98 (60)	114 (25)	135 (67)	168 (40)
INR	2.2 (0.7)	1.8 (0.2)	1.38 (0.3)	1.50 (0.1)
aPTT	48 (23)	36 (10)	45 (15)	35 (5)
Fibrinogen	189 (117)	189 (53)	220 (104)	194 (43)

*FEIBA*, factor eight inhibitor bypass activity; *R*, reaction time; *K*, coagulation time; *MA*, maximum amplitude; *A*, amplitude; *CI*, coagulation index; *LY 30*, amplitude at 30 min; *INR*, international normalized ratio; *aPTT*, activated partial thromboplastin time

There were a total of five serious adverse events in two patients resulting in death. One patient experienced postoperative complications including acute kidney failure requiring renal replacement therapy and cerebrovascular accident. The other patient had refractory bleeding diathesis and bleeding after separation from CPB requiring the administration of open-label FEIBA. Postoperatively, the patient developed a cerebrovascular accident (Table 5). These events were considered not related to the study drug.

**Table 5:** Safety and adverse events, number of events (%)

	FEIBA n = 6	Placebo n = 6
Number of patients with AEs	2	0
30-day mortality	2 (33)	0
Cerebrovascular accident	2 (33)	0
Thromboembolism	0	0
Deep vein thrombosis/pulmonary embolism	0	0
Myocardial infarction	0	0
Renal replacement therapy	1 (17)	0

*FEIBA*, factor eight inhibitor bypass activity; *AEs*, adverse events

## Discussion

In this small pilot trial evaluating the feasibility of prophylactic administration of FEIBA in a blinded fashion, we found that it was possible to comply with study procedures and adhere to the study protocol, with patients assigned to the FEIBA group receiving the active study drug in the appropriate dose, while the open-label use of rescue FEIBA was limited to refractory bleeding diathesis, according to the study algorithm.

This pilot study has several limitations including small sample size, imbalances between study groups, high variability in the requirements for blood product transfusion, and inadequate study power for efficacy endpoints. Furthermore, three patients required the use of open-label FEIBA, which could potentially negate the differences between groups in the effects of the intervention, especially if the open-label administration was provided to patients in the placebo group. However, the concern for contamination between groups was attenuated because the study was specifically designed to determine the role of the early administration of FEIBA on subsequent needs of blood product transfusion. On the other hand, it would not have been ethical to disallow the use of open-label FEIBA in the context of ongoing bleeding. To that effect, the study had equipoise for early empiric FEIBA administration in high-risk patients given with the intent of reducing potential bleeding, but not given with the intent of treating ongoing, refractory bleeding.

Due to the small sample size, the randomization did not produce equally balanced groups, with the FEIBA group resulting in an older population with more comorbidities and a longer duration of deep hypothermic circulatory arrest. Although not different between the two groups, more serious adverse events were reported in the FEIBA group. In addition, the study was not powered to detect differences in the efficacy endpoint of the total amount of blood product transfused after randomization. However, the study provides estimates of variability that will allow the calculation of the sample size for the pivotal efficacy study. We failed to find differences in the cumulative amount of blood product transfused or in the amount transfused intraoperatively or in the ICU. This is in contrast with prior findings from observational studies suggesting a relevant reduction in blood component transfusion compared to no treatment [18]. Several considerations might explain this finding, including the high variability in the volume of blood product transfused across the study population overall, the small sample size, and the relative low percentage of patients requiring transfusion despite the complex surgical procedures considered at high risk of bleeding diathesis after completion of CPB.

This trial provides important insights to inform the planning of the pivotal trial including the population selection to include patients who are more likely to require transfusion and the determination of the appropriate dose of FEIBA. Because we did not detect differences in coagulation profile between the groups after the administration of the study drug, the dose of FEIBA administered for the study may have not been adequate. It is possible that a higher dose might have resulted in more detectable changes in the coagulation profile. It is also possible that correction of the coagulation profile in the placebo group occurred by replenishing factor deficiency derived from other sources such as blood components. Due to the unbalances in baseline and intraoperative characteristics, it is challenging to further interpret differences between groups with regard to the blood product transfused. It should be emphasized that bleeding complications typically have a multifactorial origin. To what extent FEIBA treatment may reduce bleeding and transfusion requirements would require a larger clinical trial.

While this trial was not powered to detect differences between groups, future trials will need to be powered to assess comparative outcomes including cumulative volume of blood products transfused. If alternatives to packed red blood cells, fresh-frozen plasma, and platelet transfusion were available, complications could potentially be reduced. Prothrombin complex concentrates may turn out to be a lower-risk alternative to the blood products from which they are derived.

## Conclusions

We demonstrated the feasibility of a double-blind, placebo-controlled trial to test whether administration of prophylactic FEIBA decreases transfusion of blood products in patients at high risk for bleeding diathesis in cardiac surgery. Although the trial was not powered for the evaluation of efficacy, the study provides estimates of variability for the planning of a pivotal trial. We conclude that a larger trial of FEIBA administration to prophylactically replenish coagulation factors and correct post-CPB coagulopathy is feasible; however, further refinements of the study design are needed specifically with regard to the study population selection, the choice of study drug dosing, and the use of a standardized transfusion algorithm to minimize the effect of practice variability among centers. Whether a prophylactic approach might prevent refractory bleeding diathesis and reduce the need for high volumes of blood product transfusion needs to be determined in a future, larger trial.

## Data Availability

The datasets used and/or analyzed during the current study are available from the corresponding author on reasonable request.

## Abbreviations

ACT: Activated clotting time
aPTT: Activated partial thromboplastin time
CPB: Cardiopulmonary bypass
FEIBA: Factor eight inhibitor bypass activity
FFP: Fresh-frozen plasma
INR: International normalized ratio
PCC: Prothrombin complex concentrate
PLTs: Platelets
PRBCs: Packed red blood cells
SAE: Serious adverse event
TEG: Thromboelastogram
